# Role of Alcohol Metabolism in Chronic Pancreatitis

**Published:** 2007

**Authors:** Alain Vonlaufen, Jeremy S. Wilson, Romano C. Pirola, Minoti V. Apte

**Keywords:** Alcohol abuse, ethanol metabolism, ethanol-to-acetaldehyde metabolism, alcohol dehydrogenase (ADH), acetaldehyde, cytochrome P4502E1 (CYP2E1), reactive oxygen species (ROS), oxidation, pancreas, chronic pancreatitis, acute pancreatitis, alcoholic pancreatitis, acinar cell, pancreatic stellate cells (PSCs), fatty acid ethyl esters (FAEEs), genetic factors, genetic polymorphisms

## Abstract

Alcohol abuse is the major cause of chronic inflammation of the pancreas (i.e., chronic pancreatitis). Although it has long been thought that alcoholic pancreatitis is a chronic disease from the outset, evidence is accumulating to indicate that chronic damage in the pancreas may result from repeated attacks of acute tissue inflammation and death (i.e., necroinflammation). Initially, research into the pathogenesis of alcoholic pancreatitis was related to ductular and sphincteric abnormalities. In recent years, the focus has shifted to the type of pancreas cell that produces digestive juices (i.e., acinar cell). Alcohol now is known to exert a number of toxic effects on acinar cells. Notably, acinar cells have been shown to metabolize alcohol (i.e., ethanol) via both oxidative (i.e., involving oxygen) and nonoxidative pathways. The isolation and study of pancreatic stellate cells (PSCs)—the key effectors in the development of connective tissue fibers (i.e., fibrogenesis) in the pancreas—has greatly enhanced our understanding of the pathogenesis of chronic pancreatitis. Pancreatic stellate cells become activated in response to ethanol and acetaldehyde, a toxic byproduct of alcohol metabolism. In addition, PSCs have the capacity to metabolize alcohol via alcohol dehydrogenase (the major oxidizing enzyme for ethanol). The fact that only a small percentage of heavy alcoholics develop chronic pancreatitis has led to the search for precipitating factors of the disease. Several studies have investigated whether variations in ethanol-metabolizing enzymes may be a trigger factor for chronic pancreatitis, but no definite relationship has been established so far.

The pancreas is a gland that secretes digestive juices which are carried to the small intestine by the biliary system, which consists of the gall-bladder and a network of ducts. When the pancreas becomes inflamed, its digestive enzymes leak out and begin to attack the pancreas itself. These enzymes cause damage that results in swelling of tissues and blood vessels. There are two forms of inflammation of the pancreas (i.e., pancreatitis). Acute pancreatitis occurs when the pancreas suddenly becomes inflamed but then improves. Chronic pancreatitis (CP) is a progressive inflammatory disease leading to irreversible destruction of the pancreas. It is characterized by a spectrum of symptoms ranging from pain—the cardinal initial symptom in most cases— to maldigestion and diabetes. The major cause (i.e., etiology) of CP is alcohol abuse. It also is associated with genetic mutations (i.e., hereditary CP), autoimmunity, excessive production of the parathyroid hormone (i.e., hyperparathyroidism), and a type of pancreatitis seen in tropical countries (i.e., tropical pancreatitis). In a certain number of cases, CP remains etiologically undetermined (i.e., idiopathic CP) (Steer et al. 1993). The reported incidence of the disease varies widely among countries, and it remains unclear whether this is attributable to genuine regional differences or to lack of standardized diagnostic criteria.

Epidemiological studies and animal experiments suggest that alcohol, per se, is not sufficient to induce the disease. As a matter of fact, less than 10 percent of heavy alcohol users (180 g/day or about 15 drinks/day for 10 to 15 years) eventually develop clinically overt alcoholic pancreatitis. Researchers have analyzed several predisposing factors, such as the amount and pattern of drinking, smoking, dietary habits, and genetic mutations— particularly those of alcohol-metabolizing enzymes—but none of these factors has been firmly linked to the development of alcoholic CP (Ammann 2001).

Several theories about how alcohol might lead to pancreatic disease have emerged over the past decades. Whereas early work had predominantly focused on the effects of alcohol on the muscle at the surface of the first part of the small intestine (i.e., duodenum), which controls secretions from the liver, pancreas, and gallbladder into the duodenum (i.e., the sphincter of Oddi), and on the pancreatic ducts (see Figure 1), attention has shifted over the past decade to the influence of alcohol on the clusters of secretory cells (i.e., acini) that produce pancreatic juice containing digestive enzymes. Studies with acini or pancreatic acinar cells grown in the laboratory (i.e., cultured cells) have established the ability of the pancreas to metabolize alcohol via oxidative and nonoxidative pathways and have provided new insights into the toxic effects of alcohol and the byproducts of its metabolism (i.e., metabolites) on the gland. Furthermore, a new era in the understanding of the pathophysiological mechanisms of scar tissue formation in the pancreas (i.e., pancreatic fibrosis) has dawned with the recent identification and culture of pancreatic stellate cells (PSCs), the key effector cells in fibrogenesis. Of particular interest is the finding that these cells have the capacity to metabolize alcohol.

This article reviews past theories and current knowledge about the pathophysiology of chronic alcoholic pancreatitis, with particular emphasis on alcohol metabolism by acinar and stellate cells and on the toxic effects of alcohol and its metabolites on these cells.

## Effects of Alcohol on the Pancreas

It now is generally accepted that alcoholic acute and chronic pancreatitis are the same disease at different stages. Repeated episodes of tissue inflammation and death (i.e., necroinflammation) in the pancreas lead to periductular obstructive scarring and protein plug formation and eventually extensive fibrosis (i.e., necrosis–fibrosis sequence). This sequence is further supported by the fact that patients with frequent episodes of acute pancreatitis progress more rapidly to chronic disease (Ammann and Muellhaupt 1994).

### Effect of Alcohol on the Sphincter of Oddi

Initial research on the effects of alcohol on the pancreas focused on sphincter of Oddi activity. This work was based on so called “sphincteric theories” aiming to implicate reflux of the gall-bladder and bile ducts (i.e., biliary tract) or duodeno-pancreatic reflux as the causative factor in alcoholic pancreatits. Several human studies yielded conflicting results with reports of both decreased and increased sphincter of Oddi activity upon ethanol exposure (Apte et al. 1998*a*). However, the latter phenomenon—of ethanol exposure causing spasms in the sphincter of Oddi—has been given more credit by recent evidence in animals (Sonoda et al. 2005) and by the fact that pancreatic secretion is decreased after acute alcohol intake in humans (Hajnal et al. 1990).

### Effects of Alcohol on Small Ducts

Another theory states that alcohol affects the character of pancreatic fluid to favor the formation of protein plugs and stones. Contact of the stones with the ductal epithelial cells potentially could lead to ulceration, scarring, further obstruction, and finally atrophy and fibrosis. This hypothesis is supported by the findings that alcohol (1) reduces pancreatic secretions (as stated earlier); (2) leads to increased viscosity of pancreatic secretions; (3) decreases citrate concentration in pancreatic juice, a known predisposing factor for crystal formation; and (4) produces proteins thought to increase stone formation, such as pancreatic stone protein (PSP) and glycoprotein-2 (GP-2) (Stevens et al. 2004; Apte et al. 1997).

However, it remains difficult to prove whether ductal stones are a cause or an effect of CP. It is possible to imagine that the formation of protein plugs could contribute to small-duct obstruction. A prototypical example of such a disease mechanism is represented by cystic fibrosis, which is known to enhance the viscosity of pancreatic secretions—hence promoting stone formation—and significantly increasing the risk of CP in affected individuals.

## Direct Toxic Effects of Ethanol on Acinar Cells

**Figure 2:**
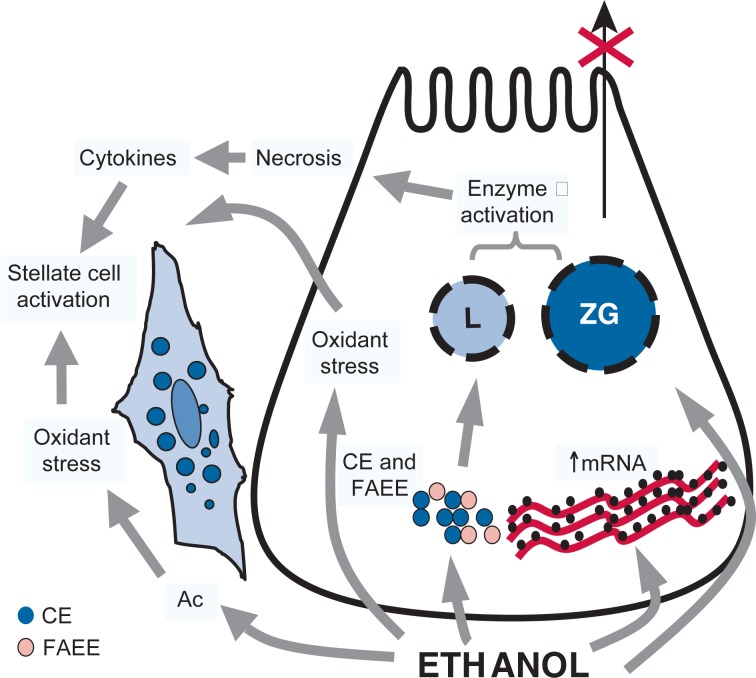
The Figure depicts an overall hypothesis for the pathogenesis of alcoholic pancreatitis. It is postulated that ethanol, its metabolites, and oxidant stress exert a number of toxic effects on pancreatic acinar cells, which predispose the gland to autodigestive injury. These include the following:
Destabilization of lysosomes (L) and zymogen granules (ZG). This destabilization is mediated by oxidant stress; cholesteryl esters (CEs), which are known to accumulate in the pancreas during ethanol consumption; and fatty acid ethyl esters (FAEEs), which are nonoxidative metabolites of alcohol.Increased digestive and lysosomal enzyme content attributed to increased synthesis (increased mRNA) and impaired secretion.These changes sensitize the cell such that in the presence of an appropriate trigger/co-factor overt injury is initiated (alcoholic acute pancreatitis). Cytokines released during alcohol-induced necroinflammation activate pancreatic stellate cells (PSCs). In addition, PSCs are activated directly by ethanol, most likely via its metabolism to acetaldehyde (Ac) and the subsequent generation of oxidant stress. Activated PSCs then synthesize excess amounts of extracellular matrix proteins leading to pancreatic fibrosis.

Given the failure of the sphincteric and ductular obstruction theories to fully explain the pathogenesis of alcoholic pancreatitis, the attention of researchers has shifted over the past 10 years toward the acinar cells, the most abundant cells in the pancreas. The acinar cell constitutes an “enzyme factory” that produces millions of digestive enzyme molecules every day. These enzymes are produced as inactive precursors, packed into stable vesicles (i.e., zymogen granules), and segregated from cellular componenets that can break down other cellular components (i.e., lysosomal enzymes) in order to avoid premature activation. It has been consistently shown in various animal models that one of the first events in acute experimental pancreatitis consists of the premature activation of digestive enzymes within the acinar cell by co-segregation of zymogen granules with lysosomal enzymes, particularly cathepsin B. This and other toxic effects of ethanol on acinar cells are depicted in Figure 2 and described below.

### Effect of Ethanol on Pancreatic Enzymes

Experimental studies have shown that alcohol consumption leads to an increased amount of digestive (trypsin, chymotrypsin, and lipase) and lysosomal (cathepsin B) enzymes and concurrently increases lysosome and zymogen granule fragility. This is thought to facilitate contact between lysosomal and digestive enzymes, thereby predisposing the cell to breakdown by its own enzymes (i.e., autodigestion). The effect of alcohol on lysosomal fragility may be mediated by cholesterol esters and toxic substances that accumulate in the pancreas after chronic alcohol intake (i.e., fatty acid ethyl esters [FAEEs]). It also has been shown that ethanol administration in rats decreases levels of GP-2, a glycoprotein known to stabilize the membranes of zymogen granules (Apte et al. 1997).

Recent work by Wang and colleagues (2006) indicates that chronic ethanol consumption in rats decreases controlled cell death (i.e., aptoptosis) and increases the production of cathepsin B, thereby potentially promoting necrotic changes in the pancreas.

### Metabolism of Ethanol by Acinar Cells

Toxic metabolites of ethanol are known to have adverse effects on several organs in the body. Researchers have therefore focused on the capacity of the functional cells of the pancreas to metabolize alcohol. Based on studies in the liver, it is well known that the metabolism of ethanol—via oxidative and nonoxidative pathways—generates the toxic metabolites acetaldehyde and FAEEs, respectively. The oxidative pathway is catalyzed predominantly by the enzyme alcohol dehydrogenase (ADH), with relatively minor contributions by cytochrome P450 2E1 (CYP2E1) and catalase. The nonoxidative pathway of ethanol involves a chemical reaction (i.e., esterification) of ethanol with fatty acids, resulting in the formation of FAEEs. The enzymes that catalyze FAEE production (i.e., FAEE synthases) still need to be fully characterized, but putative candidates include carboxylester lipase and triglyceride lipase (Wilson and Apte 2003).

Using cultures of acinar cells or isolated pancreatic acini, researchers now have firmly established that pancreatic acinar cells are capable of metabolizing ethanol via both the oxidative and the nonoxidative pathways (Haber et al. 1998, 2004; Gukovskaya et al. 2002). Concerning the oxidative pathways, the predominant variation (i.e., isoform) of ADH in acinar cells appears to be ADH3, a nonsaturable form^1^ of the enzyme with low affinity and a high *K*_m_ for ethanol. CYP2E1 has been described in human and rat pancreas. As previously observed in the liver, its expression is inducible in the pancreas of alcohol-fed rats.

Regarding the nonoxidative pathway of ethanol metabolism, the pancreas appears to accumulate more FAEEs after ethanol abuse than any other functional—as opposed to structural—organ. Although FAEEs may be transported to the pancreas from the liver via the circulation, it now has been shown that these compounds (predoniminantly ethyl pamitate) are synthesized in the pancreas itself. Notably, FAEE synthase activity reportedly is higher in the pancreas (3.5- to 10-fold) than in the liver (Wilson and Apte 2003).

Werner and colleagues (2002) have tried to establish a link between the two metabolic pathways for ethanol. They have reported that in the presence of inhibitors of oxidative metabolism, the generation of FAEEs in isolated acini is increased compared with FAEE generation in the absence of inhibitors. The authors also have shown that in vivo infusion of ethanol with inhibitors of oxidative metabolism leads to increased FAEE accumulation in the rat pancreas.

As a consequence of the above studies, the rates of ethanol metabolism via the oxidative and the nonoxidative pathways recently have been compared in rat pancreatic acini incubated with the same concentration of alcohol (100 micromoles^2^ [μM]). The rate of oxidative metabolism was 21-fold higher compared with the rate of nonoxidative metabolism (Haber et al. 2004; Gukovskaya et al. 2002). This finding does not diminish the importance of the nonoxidative metabolism of ethanol in the pancreas. Indeed, concentrations of the products of this pathway (FAEEs) in the pancreas (in the range of 50 μM) have been shown to be sufficient to cause pancreatic injury (Haber et al. 1993).

### Effects of Toxic Metabolites of Alcohol

Acetaldehyde and reactive oxygen species (ROS) (products of ethanol oxidation) as well as FAEEs all have been shown to cause deleterious effects on the pancreas. At high concentrations, acetaldehyde induces morphological alterations in the rat and dog pancreas (Majumdar et al. 1986; Nordback et al. 1991). Furthermore, it inhibits stimulated secretion—by the enzyme cholecystokinin—from isolated rat pancreatic acini. This phenomenon is thought to be the result of acetaldehyde interfering with the binding of cholecystokinin to its cellular communication sites (i.e., receptors) and to a disruption in the functioning of cell components (i.e., microtubulues) responsible for transporting intracellular compartments.

ROS are highly reactive compounds that potentially are harmful to cell membranes, intracellular proteins, and DNA. Oxidant stress results from an imbalance between the production of ROS and the defense mechanisms (the antioxidant glutathione and the enzymes glutathione peroxidase, superoxide dismutase, and catalase) within the cell. This may be the result of (1) the generation of ROS during oxidation of ethanol by CYP2E1 and (2) acetaldehyde-induced depletion of the ROS scavenger glutathione. Oxidant stress is thought to destabilize zymogen granules and lysosomes, potentially increasing the risk of intra-acinar activation of digestive enzymes (Apte et al. 2003).

As noted earlier, FAEEs are produced by the nonoxidative metabolism of ethanol. These compounds have been shown to accumulate in rat pancreas and to cause acinar cell damage in vitro by inducing lysosomal fragility (Haber et al. 1993). Infusion of FAEEs in rats leads to the activation of trypsinogen, the precursor to the enzyme trypsin, and subsequent morphological alterations consistent with acute pancreatitis. Furthermore, in vivo studies have shown that FAEE infusion leads to increased deposition of material that is part of the tissue but not part of any cell (i.e., extracellular matrix) in the rat pancreas, a feature that might be relevant to the development of alcohol-induced fibrosis (Lugea et al. 2003).

Studies to date suggest that FAEEs exert their toxicity by a number of different mechanisms, including (1) direct interaction with cellular membranes; (2) promotion of cholesteryl esters, which accumulate in the rat pancreas after chronic ethanol intake and destabilize lysosomal membranes; (3) activation of transcription factors^3^ known to regulate the production of molecules involved in the inflammatory response (i.e., cytokines); (4) the release of free fatty acids by the break down (i.e., hydrolysis) of FAEEs, a process thought to contribute to damage in cellular structures called mitochondria; and (5) disruption of calcium homoeostasis in pancreatic acinar cells.

Recent research has focused on cell signaling events as mediators of ethanol-induced pancreatic injury. Gukovskaya and colleagues (2002) have shown that ethanol and its metabolite acetaldehyde regulate the transcription factors nuclear factor κB (NFκB) and AP-1 in acinar cells. These signaling molecules are implicated in cytokine production. Recently, more light has been shed on another mechanism of FAEE toxicity in the cell––the disruption of calcium homeostasis in acinar cells. In vitro work has shown that the FAEE palmitoleic acid ethyl ester induces a significant increase of calcium in the substance filling the acinar cell (i.e., acinar cell cytoplasm). This is caused by dysfunction of membrane-bound enzymes called ATPases that hydrolyze the ester to its free fatty acid, palmitoleic acid. Palmitoleic acid appears to disrupt the mitochondrial oxidative chain, leading to deficient production of the energy-producing molecule adenosine triphosphate (ATP), calcium overload, and cell death. These recent findings describe specific pathways that might be targeted therapeutically to reduce the noxious effects of alcohol on the pancreas (Criddle et al. 2006).

## Pancreatic Stellate Cells

One of the key features in the microscopic examination of alcoholic CP is pancreatic fibrosis. Decisive advances in our understanding of pancreatic fibrosis have been made by the ability to isolate and culture PSCs (Apte et al. 1998*b*). Their critical role in pancreatic fibrogenesis now is firmly established.

Stellate cells are located in the vicinity of the base or bottom part (i.e., basal aspect) of acinar cells. In the normal pancreas, stellate cells exist in an inactive (i.e., quiescent) state, identifiable by the presence of vitamin A–containing fat (i.e., lipid) droplets in the cytoplasm and positive immunostaining^4^ for proteins that make up the cell’s structure (i.e., cytoskeletal proteins), such as desmin and glial acidic fibrillary protein. In health, these cells are thought to play a role in extracellular matrix turnover via their ability to synthesize extracellular matrix proteins as well as matrix-degrading enzymes (matrix metalloproteinases [MMPs]). Evidence from in vitro and in vivo studies indicates that, during pancreatic injury, PSCs can be activated by profibrogenic growth factors, such as transforming growth factor-beta (TGF-β); proliferative growth factors such as platelet-derived growth factor (PDGF); proinflammatory cytokines; and oxidant stress (Apte and Wilson 2003). This state of activation is characterized by the loss of vitamin A droplets, the production of alpha smooth muscle actin (α-SMA, a cytoskeletal protein), and increased production of extracellular matrix proteins such as collagens I and III, fibronectin, and laminin. Of note, activated PSCs also produce increased amounts of MMP2 (known to degrade the structural protein collagen, thereby facilitating the deposition of fiber-forming collagen observed in pancreatic fibrosis) and of its inhibitor, tissue inhibitor of metalloproteinase 2 (TIMP2).

Of particular interest to the pathogenesis of chronic alcoholic pancreatitis is the finding that PSCs are activated by exposure to physiologically relevant concentrations of ethanol (10 and 50 mM [i.e., 1 to 2 and 8 to 10 standard drinks], respectively), corresponding to blood alcohol concentrations encountered during social drinking and heavy alcohol consumption respectively) or acetyldehyde (150 and 200 mM) as assessed by increased α-SMA expression and collagen synthesis. It also has been shown that exposure of PSCs to ethanol and acetaldehyde in vitro increases the secretion of MMP2, which may contribute to pancreatic fibrosis, as described above.

Interestingly, stellate cells have been shown to exhibit ADH (isoform 1) activity that is induced by exposure to ethanol at a concentration of 50 mM. Inhibition of ADH1—and the conversion of ethanol to acetaldehyde—abolished PSC activation, suggesting that the effect of ethanol on the cells is mediated by its oxidation to acetaldehyde. Both ethanol and acetaldehyde caused oxidant stress in cultured PSCs. Incubation of PSCs with ethanol or acetyldehyde in the presence of the antioxidant vitamin E prevented PSC activation (Apte et al. 2000). Taken together, the above findings suggest that ethanol-induced PSC activation is mediated via the metabolism of ethanol to acetaldehyde and the subsequent generation of oxidant stress within the cells.

Another pathway of stellate cell activation by alcohol might be related to its common metabolism with compounds chemically related to vitamin A (i.e., retinoids). As mentioned earlier, storage of vitamin A is a key feature of quiescent stellate cells both in the liver and in the pancreas. Importantly, quiescence of stellate cells in the liver has been shown to depend on sufficient concentrations of the retinol metabolite retinoic acid in the liver. It has been demonstrated in the liver that ethanol competitively inhibits retinol metabolism by retinol dehydrogenase and aldehyde dehydrogenase because these enzymes can degrade both ethanol and retinol. This is supported by the recent finding that the inhibitory effect of vitamin A on PSC activation partially can be reversed in the presence of ethanol (McCarroll et al. 2006). Thus, vitamin A may represent a potential therapeutic strategy for chronic pancreatitis, given its ability to inhibit PSC activation and synthesis of extracellular matrix proteins.

### Role of Genetic Variations in Ethanol-Metabolizing Enzymes

It is evident from the above that alcohol exerts constant and toxic effects on different cellular compartments in the pancreas, predisposing the gland to autodigestion, necroinflammation, and fibrosis. As stated earlier, the risk of alcoholic acute and chronic pancreatitis increases with increasing intake of ethanol. However, only a minority of heavy drinkers will develop clinically evident pancreatitis, suggesting that additional co-factors are required to trigger overt disease. Efforts have been made to identify risk factors over the past 20 years, including dietary factors, smoking, amount and type of alcohol consumed, pattern of drinking, lipid intolerance, and inherited factors.

Given that pancreatic ethanol metabolism appears to play a significant role in the pathophysiology of pancreatitis, it is not unreasonable to speculate that variations (i.e., polymorphism) in ethanol-metabolizing enzymes might be a modifying factor of the disease. Researchers have conducted numerous case–control studies in an attempt to link polymorphisms of ethanol-metabolizing enzymes to alcohol-related pancreatic damage. Such studies of potential risk factors for alcoholic pancreatitis ideally should compare alcoholics without pancreatic disease with alcoholics displaying pancreatic injury, but this has not always been the case.

Frenzer and colleagues (2002) conducted a case–control study comparing the genes for *ADH2*, *ADH3*, *ALDH2*, and *CYP2E1* in 57 Caucasian patients with alcoholic cirrhosis, 71 patients with alcoholic CP, 57 alcoholics without any apparent organ damage, and 200 healthy blood donors. The authors were able to detect a definite association between the genetic variation ADH3*2/*2 and possibly ADH2*1/*1 and alcoholic cirrhosis. However, no association was found between the above polymorphisms and CP.

An earlier case–control study, conducted in Chinese patients, analyzed *ADH2*, *ADH3*, *ALDH2*, and *CYP2E1* genes in patients with acute alcoholic pancreatitis, in alcoholic patients without organ damage, in patients with pancreatic disease of nonalcoholic origin, and in patients with alcoholic liver disease. This study found that the frequency of the ALDH2*1 gene variant (i.e., allele) was significantly increased in alcoholics compared with nonalcoholic control subjects. No significant difference in terms of polymorphism between alcoholic pancreatitis and alcohol abuse without organ damage could be found. It must be noted, however, that the control group of heavy drinkers without organ damage was small (19 patients) and may not be a representative cohort (Chao et al. 1997).

A recent case–control study conducted by Verlaan and colleagues (2004) compared *ADH3* and *CYP2E1* polymorphism in 82 patients with alcoholic CP, 21 patients with hereditary pancreatitis, 39 patients with idiopathic pancreatitis, 93 alcoholic and 128 healthy control subjects. All subjects were of Caucasian origin. No significant difference between patients with CP of various etiologies and controls was observed, although the diagnosis of CP was not based on uniform criteria. Nonetheless, the researchers reported a trend to a higher frequency of a particular allele for *CYP2E1* (i.e., the intron 6D allele) in patients with alcoholic CP compared with healthy or alcoholic control subjects.

Most recently, a Japanese study (Miyasaka et al. 2005) has reported a promising association between the risk of developing pancreatitis and a polymorphism of the gene for one of the candidate FAEE synthase enzymes (i.e., carboxyl ester lipase [*CEL*]) and the risk of developing alcoholic pancreatitis. The authors examined a polymorphism (the VNTR polymorphism) in the coding region of the *CEL* gene. The significance of this polymorphism is not yet clearly defined, but it has been suggested that it might influence protein stability and/or secretion. The study included a population of 100 alcoholic subjects with CP, 52 alcoholic subjects, 50 nonalcoholic pancreatitis patients, 96 patients with elevated levels of lipids in the bloodstream (hyperlipidemia), and 435 control subjects. The frequency of the usual (wild-type) gene was significantly diminished in patients with alcoholic pancreatitis compared with the other groups, including alcoholic subjects without organ damage.

In summary, current data fail to establish an unequivocal link between any polymorphism of ethanol-oxidizing enzymes and the risk of alcoholic pancreatitis. With respect to the nonoxidative pathway of ethanol metabolism, the polymorphism of *CEL* is of interest, but the functional significance of this polymorphism is yet to be defined.

## Conclusion

There now is sufficient evidence that the pancreas has the capacity to metabolize ethanol via both oxidative and the nonoxidative pathways. The resulting metabolites and their byproducts (oxygen radicals) exert a toxic effect on the pancreas, leading to acute and chronic changes, but the susceptibility factor that triggers overt disease remains to be identified.

The capacity of PSCs to metabolize alcohol and to become activated under the influence of acetaldehyde and oxidant stress are key features with respect to the role of these cells in alcoholic pancreatic fibrosis. Further studies designed to characterize the metabolism of ethanol by PSCs via the oxidative and nonoxidative pathways are awaited.

## Figures and Tables

**Figure 1:**
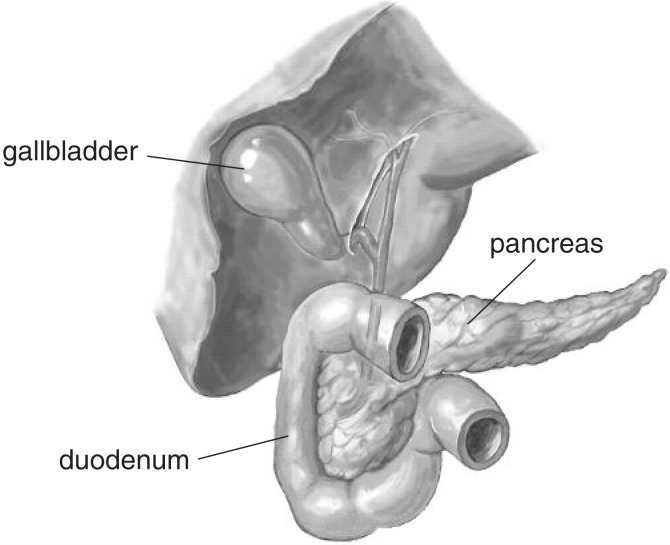
Illustrated are the pancreas, gallbladder, and duodenum.

## Footnotes

1 A nonsaturable form of an enzyme cannot be saturated (i.e., its activity is not limited) even by very high concentrations of the substrate (i.e. alcohol).

2 A micromole represents a concentration of 1/1,000,000 (one millionth) molecular weight per liter (mol/L).

3 Transcription factors are proteins involved in the process that uses DNA to make proteins.

4 Immunostaining refers to any use of an antibody-based method to detect a specific protein in a sample.
